# Enhancing QoS of Telecom Networks through Server Load Management in Software-Defined Networking (SDN)

**DOI:** 10.3390/s23239324

**Published:** 2023-11-22

**Authors:** Khawaja Tahir Mehmood, Shahid Atiq, Muhammad Majid Hussain

**Affiliations:** 1 Department of Electrical Engineering, Khwaja Fareed University of Engineering & Information Technology, Rahim Yar Khan 64200, Pakistan; shahid.atiq@kfueit.edu.pk; 2 School of Engineering and Physical Sciences, Heriot-Watt University, Edinburgh EH14 4AS, UK

**Keywords:** DASLM, data transfer rate, end-to-end user delay, HTTP, maximum available bandwidth, QoS, server load management, SDN

## Abstract

In the modern era, with the emergence of the Internet of Things (IoT), big data applications, cloud computing, and the ever-increasing demand for high-speed internet with the aid of upgraded telecom network resources, users now require virtualization of the network for smart handling of modern-day challenges to obtain better services (in terms of security, reliability, scalability, etc.). These requirements can be fulfilled by using software-defined networking (SDN). This research article emphasizes one of the major aspects of the practical implementation of SDN to enhance the QoS of a virtual network through the load management of network servers. In an SDN-based network, several servers are available to fulfill users’ hypertext transfer protocol (HTTP) requests to ensure dynamic routing under the influence of the SDN controller. However, if the number of requests is directed to a specific server, the controller is bound to follow the user-programmed instructions, and the load on that server is increased, which results in (a) an increase in end-to-end user delay, (b) a decrease in the data transfer rate, and (c) a decrease in the available bandwidth of the targeted server. All of the above-mentioned factors will result in the degradation of network QoS. With the implementation of the proposed algorithm, dynamic active sensing server load management (DASLM), on the SDN controller, the load on the server is shared based on QoS control parameters (throughput, response time, round trip time, etc.). The overall delay is reduced, and the bandwidth utilization along with throughput is also increased.

## 1. Introduction

**Figure 1:**
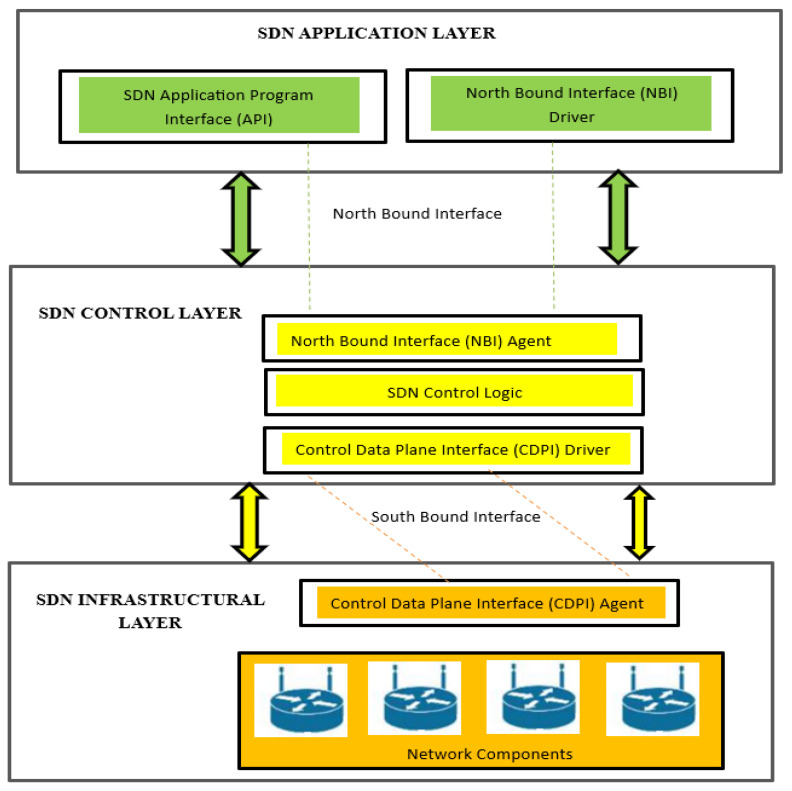
Layered structural model of SDN (software-defined networking).

Owing to recent advancements in cloud computing, big data applications, and complex network architecture with machine learning applications, legacy networks cannot fulfill the demands of system administrators and network users. Currently, the primary goal of every network designer is to provide fast and reliable data transformers (wired or wireless). The other important aspect is maintaining QoS, that is, achieving better throughput, greater bandwidth, and less latency delay. Virtualization reduces the number of physical components in the network, which can result in energy-efficient and maintenance-free systems. A network system that provides all of the applications mentioned above is a Software-Defined Networking (SDN). At the same time, SDN is a centralized-based [1] virtual control system that provides an entire view of the network underlying its control layer. SDN reduces the extra burden by separating the data and control layers from the network components [2] and performs a virtual control process using an SDN controller based on the logical instructions provided by the application layer. A research article [3] showed the performance-based results of a heavily loaded network environment consisting of 256 servers for data modulation by testing different network systems. They concluded that SDN performance in data modulation was 47% greater than the legacy network. In Hadoop applications [4], SDN outperforms legacy networks regarding controllability, scalability, and flexibility in normal forwarding modes. SDN with a centralized controlled technique can provide better controllability, scalability, and reliability than traditional physical networks. Currently, all networking architectures employ software (IP—version: 6, BGP—version: 4, MPLS—version: 3, VMware—version: 17.5.0, etc.) for data management and transformation [5]. All protocols mentioned above can be easily manipulated in an SDN virtual environment; with this flexibility, SDN is becoming a vital networking force [6,7]. The QoS parameters are the primary deterministic performance tools in tele-traffic engineering. The QoS results of SDN with separate data and control layers [8,9] are far superior to legacy networks. Figure 1 shows the layered structure of an SDN. As previously mentioned, the SDN consists of three layers, namely, the application, control, and infrastructural layers.

Five important parameters exist to accomplish a complete view of SDN operation: the SDN application plane, control plane, data plane, northbound interface/northbound interface (NBI) agent, and southbound interface/control data plane interface (CDPI) [10,11]. The user interacts with the SDN network through the application layer by providing instructions to the controller of the SDN in simple logic, depending on which type of controller is selected to manage the flow of network components. The northbound interface is used to connect the application and control layers. With the help of the NBI agent, the controller in the second layer coordinates with the application layer and controls the network components (switches, routers, etc.) according to the instructions provided by the designer in the application layer. The southbound-CDPI correlates with the control and data planes. The network component flow table is managed by an SDN controller using a CDPI agent. It informs the user of the flow result and QoS parameters with the help of NBI agents for further system data modulation.

### Motivations and Significance of Research Technique

In this research, the proposed technique (DASLM) provides the following features:

(a) By applying the DASLM approach, there is less end-to-end latency delay, maximum throughput, and less queuing delays by avoiding elephant flows, equal load management, and efficient use of bandwidth can be achieved.
(b) Bandwidth enhancement is obtained using the proposed (DASLM) technique.
(c) In the case of large networks (more switches, hosts, data center servers), the single controller could become loaded, exhausted, and lead to a single-point failure. The solution mentioned in different research techniques, as discussed in Section 2.1, was to use multiple controllers (in the master-salve version). The major issues in the implementation of these methods involve the following:
  (1) The compatibility of two controllers.
  (2) Providing the data center server access to both controllers.
  (3) Switches flow managed by both controllers.

To overcome this problem, the large SDN network (i.e., servers in the network having to serve HTTP requests greater than thirty thousand per second and with more than two hundred HTTP requests in queue to be processed by the respective servers) is divided into the smaller SDN networks with the controller in a logically distributed controlled environment (to overcome the compatibility issue). Each controller is responsible for their (subdivided) SDN network.

(d) The other major problem addressed in the proposed technique is the load balancing in the HTTP service provider servers by calculating the number of HTTP requests on each server and computing the server load. The HTTP request per second value (RPS) of each server is compared with the reference server load threshold (S_LT_) value (as explained in Section 3.1), which is set to a level of 1000 (HTTP requests per second) in our case. If the number of HTTP requests on the particular server has reached the (S_LT_) value, then that server is considered loaded and is removed from the available pool of servers in the SDN network, and no new HTTP request is assigned to that server until the RPS value decreases below the S_LT_ value range. The load is balanced by directing the flow of HTTP requests from the loaded server to other available servers on the following bases:

The new HTTP request flow is assigned to the server with a lower RPS load value than the list of available servers in the network.

(OR)

The new HTTP request flow is assigned to the server with less than 60 HTTP requests in the queue (considered a reference value in our case) in addition to the HTTP requests being processed by that server. 

(OR)

The new HTTP request flow is assigned to the server with a quicker response time than all other available servers in the network. This task is accomplished by sending an ARP packet to the servers. The server that responds to the controller with less latency is then forwarded the new HTTP request flow.

However, the detail about the functioning of the proposed (DASLM) algorithm is mentioned in Section 3.1.

**However, the paper structure summary is as follows:**

Section 2 discusses the literature review and compares traditional load-balancing methods with the proposed (DASLM) algorithm. Section 3 explains the methodology of the proposed technique, lab setup details, and procedural steps. Section 4 discusses the simulation results obtained in two portions: (1) without implementing the proposed algorithm (DASLM) on the SDN controller and (2) with implementing the proposed algorithm (DASLM) on the SDN controller. Each portion is simulated for two cases: (a) normal flow and (b) loaded flow, and QoS parameter comparison is performed with some traditional server load-balancing techniques to check the performance of the proposed (DASLM) algorithm. Section 5 summarizes the simulation result in conclusion with future directions.

## 2. Literature Review

### 2.1. Traditional Methods for Load Balancing in SDN Network

**Figure 2:**
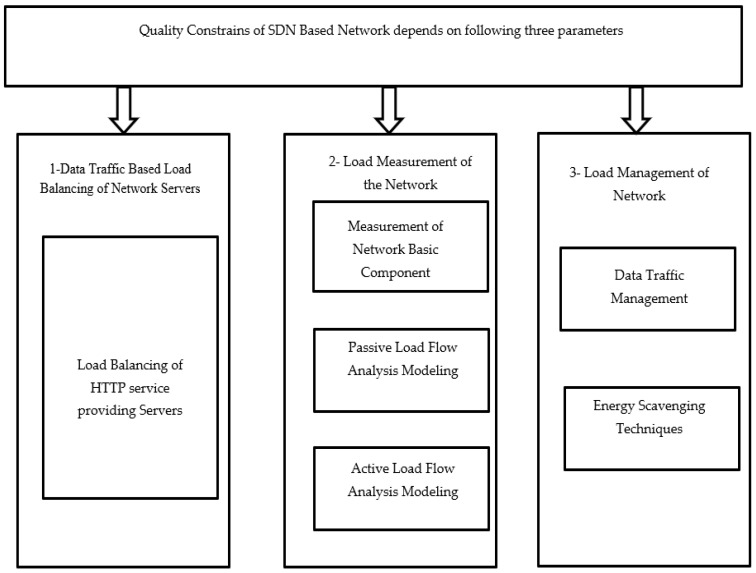
Structural view of literature review conducted in three main categories.

This section summarizes the theoretical results of the different research techniques for obtaining better QoS parameters. This portion is divided into three main categories to identify the research gap, as shown in Figure 2. The results are summarized as follows: (a) data flow (from the control plane to the data plane or vice-versa); (b) control flow (from the application layer to the control layer (and vice-versa) and the control plane to the data plane) [12]. The user interacts with the SDN network through the application layer by providing instructions to the controller of the SDN in simple logic, depending on which controllers are selected to manage the flow of network components.

#### 2.1.1. Load-Balancing Techniques of Network Servers

The network servers are connected in group form to fulfill the users’ hypertext transfer protocol (HTTP) requests. However, servers are becoming exhausted due to the ever-increasing user request load. If server load management is neglected, the specific links will be overloaded, the HTTP request will be queued, an end-to-end delay will increase, and QoS will be deterred. This scenario will also lead to the complete network’s exhaustion, eventually collapsing. Several QoS-oriented algorithms have been developed in modern-day research [1]. The authors of a research article [13] focused on the controller. Instead of using a single controller, they used master and slave versions. One controller looked after the flow, and the other managed the control signal; therefore, due to this technique, the load was shared between two controllers. This resulted in reduced end-to-end delay and improved bandwidth utilization and throughput. However, this method added complexity regarding the compatibility of both controllers while transferring load and necessary control information. Another problem associated with this technique is the high energy consumption. In a previous study [14], load balancing was performed on network switches with an additional entry of data flow, so the data packets should have led to a specific server with less migration. The major problem was that if a particular server was requested more than other servers, that particular server would be loaded, so the technique mentioned above must be revised. In a previous study [15], the authors discussed a geo-based routing protocol in which the controller forwards the data packets to the servers closest to the switch so that there is a lower cost of migration. This leads to a problem: if the specific server is close to more switches in the network compared to the other servers, then that server will be loaded, and the QoS will be compromised. In a previous study [16], the static load-balancing algorithm was used, with data traffic categorized into two groups: (a) critical time traffic and (b) non-critical time traffic. First, the critical time traffic is routed if the server is loaded with critical time traffic flow, then the non-critical time traffic is discarded, which leads to a reduction in bandwidth utilization, and the overall throughput of the network is reduced. It is based on weighted Round-Robin method. In a research article [17], the authors added a programmable middle-box to assist with the SDN controller. It calculates the load on each server and guides the controller regarding server HTTP request handling and the packet drop ratio. This helps to share the load equally among the servers, but this leads to the additional complexity of programming the middle-box and controller. The middle-box and the controller should be compatible. In a previous study [18], the authors suggested using flux function calculations to find the switching weights routing cost from the server to the switch and then routing the data packets to the corresponding servers. The major problem is that if the routing cost of a specific server is low and is already loaded, then the above-mentioned network technique is inefficient. The researchers in [19,20,21,22,23] proposed that multiple controllers are the solution to QoS routing in SDN, but they introduce more complexity. The authors of [24,25] suggested that only the load can be balanced by adding weight to the data. Data-based load balancing was performed in a previous study [26]. A research article [27] discussed web server-based load balancing. In a research article [28], the authors compared different load-balancing techniques to determine the research gap for increasing QoS in telecom networks. In a research article [29], the authors combined a content delivery network (CDN) and SDN to enhance the quality of the network. In a research article [30], the authors suggested a traditional round ribbon technique for maximizing the availability of servers. In a research article [31], the authors implemented the dynamic and static load-balancing methods and drew their fruitful effects. In a research article [32,33], the authors implemented load balancing with SDN on Campus Networks (CN).

#### 2.1.2. Measurement of Network Basic Component

**Figure 3:**
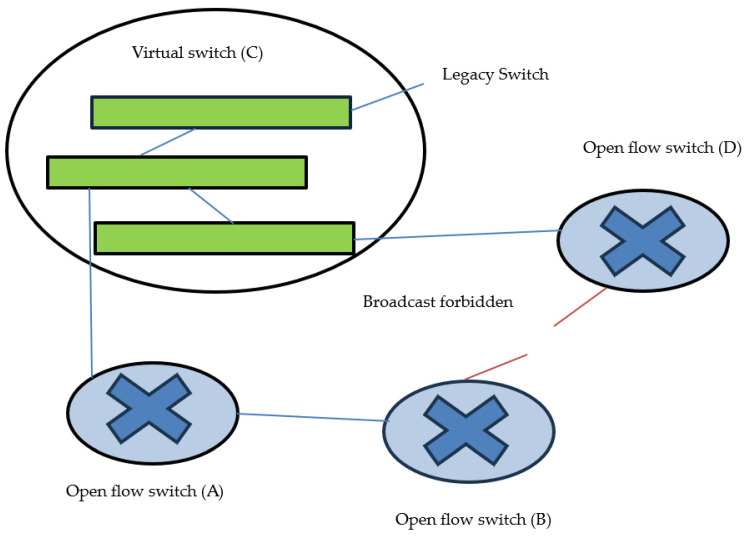
Open flow switches working with legacy switches in hybrid mode.

SDN is a centralized, controlled software-based approach that provides an abstract view of the entire network arrangement and manages its flow using a controller. The controller obtains the topological information of the underlying network devices by running protocols, such as the Link Layer Discovery Protocol (LLDP) and Spanning Tree Protocol (STP) [34]. In these protocols, the controller broadcasts the message (pack-out) to all switches in the network. The switches that receive the message (pack-out) forward the message (forward-out) to all other switches directly connected to them. In this way, all switches receive broadcast messages. All switches respond to the controller with a message (pack-in) that includes information about the switch and the other switches to which this switch is directly connected. This is how the controller of SDN creates an abstract view of network topology and gives the whole network a picture for scalability purposes to the application layer using a northbound interface [35]. In a research article [6], the researchers mentioned that changing all of the network switches (legacy) to virtual switches is not practically suitable, so it is better to make them operate in hybrid mode. The operation mode of the hybrid method is illustrated in Figure 3. In a research article [36], the method discussed (Prog-ME) provides a statistical flow graph of each node of the network, and the average load on a particular node can be seen using this method. In a research article [37], the technique discussed (Open-TM) was used to determine the average load on each node and provide better results than the above-mentioned method. A research article [38] addressed the technique (I-Stamp) used to calculate the flow of each node by creating a matrix. The results obtained using this method were far more accurate than those obtained using the aforementioned method. However, the problem with this method is that the number of flows (two or more) between two identical switches is given a single entry in the matrix; therefore, the overall load calculations with this method do not provide accurate results. In a research article [39], the traffic flow in data centers was performed using Top-of-Rack (ToR) switches which are challenging to implement in a network system. A research articles [40,41,42] discussed several flow methods to judge how many controllers must be required to fulfill the network administrator’s demand and avoid the controller side’s delay.

#### 2.1.3. Passive Load Flow Analysis Modeling

A network load is divided into two main categories: (1) passive flow and (2) active flow. In this section, several techniques that are regarded as passive flow analyses are discussed. In a research article [43], the net flow developed by Cisco was discussed, providing an average graph of the network load instead of each flow. A research article [44,45] discusses S-flow and J-flow, indicating the con-troller about each network flow, which is excellent for a network with fewer network components and loads. Under loaded conditions, the S and J flow method can be irritating. In a research article [46], the polling method showed only the traffic graph if the user requests the northbound interface by instructing the application layer. A research article [47] discussed hash algorithm methods involving extensive matrix calculations for traffic detection. In a research article [48], the dream physical project was introduced to determine the flows that must be mentioned by the controller to the application layer using NBI. Not every flow must be considered to avoid loading the controller.

#### 2.1.4. Active Load Flow Analysis Modeling

Dynamic load flow analysis refers to traffic flow estimation by considering each flow rather than the average. The traffic estimation algorithm was developed in a previous study [49]; however, the researchers used fewer network components and a limited flow. This method can be applied to a specific scenario but not in real-time loaded conditions. A research article [50] selected the traffic detection model by considering only the active switch (switches with more data transfer are considered operational, and the rest are considered in sleep mode). This applies to small network systems. A research article [51] discussed several methods for determining flow calculation errors. In ref. [52], the veri-flow method is discussed, which can be placed between the control layer and data layer, providing the liberty that not all of the load flow must be managed by the controller but instead driven by the veri-flow. However, if there is no information in the veri-flow database regarding new data flow, the controller manages this new data traffic.

#### 2.1.5. Data Traffic Management

**Figure 4:**
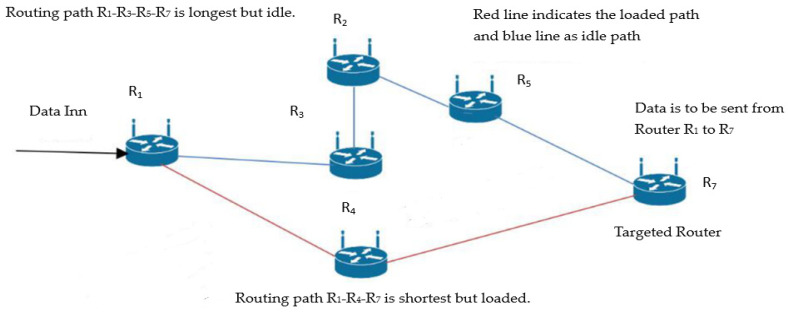
Scenario of elephant flow formation in multipath routing.

The multipath routing scheme must be optimized to achieve better controllability of the data traffic. In a research article [53], one significant and straightforward problem related to Equivalent Multipath Routing (EMR) was the formation of elephant flows. An example formulation of the elephant flow is shown in Figure 4. In normal circumstances, for fast data transformation, the shortest path between two routers is selected for data transformation. However, if that path is loaded and no alternate route is chosen, the queue and delays will increase, and the overall throughput with bandwidth will decrease, resulting in reduced quality constraints. In refs. [54,55,56,57], several logics were developed to judge elephant flow scenarios. However, they involved extensive iteration of complex logic, which can produce several syntax errors and are challenging to manage. In ref. [58], the data were routed through different routers by considering the shortest path but with the addition of the timer. If the flow of data(packets) from one router to another occurs before the timer exists, the flow is considered normal. However, if the above-mentioned condition is not satisfied, the flow is regarded as an elephant flow, and the packet is rejected and requested to be sent again. In ref. [59], the priority was selected for each flow. The heavy priority flow is treated by timer management, and the rest is treated by a common EMR technique [60]. In refs. [61,62], link state optimization was adopted to overcome the aforementioned issues. If the shortest path is loaded, the data are shifted to the router with fewer packet forwarding requests under the supervision of the SDN controller.

#### 2.1.6. Energy Scavenging Techniques

Today, the necessity of the hour is to save energy because we are facing a severe energy crisis. In a research article [63,64], the overall energy consumption by the network components (routers, switches, etc.) is 5% of the total energy consumed in everyday life in developed countries and is assumed to be 10% by the end of the year 2020, owing to the revolutionized advancement in the field of networking. In a research article [65], several hit-and trial-based methods are adopted to use fewer switches to save energy. In refs. [66,67], the energy scavenging technique makes the switches in standby mode; this factor saves 35% [68] of the energy.

### 2.2. Comparisons of the Proposed Algorithm (DASLM) with Traditional Load-Balancing Methods

Table 1 compares traditional load-balancing techniques with the proposed algorithm (DASLM).

## 3. Research Methodology

This section is further subdivided into two parts (the theoretical background and the methodology of the proposed technique).

### 3.1. Foundational Theoretical Background

Before proceeding to the implementation of the proposed research technique, the foundation theory of the proposed method in the flow steps is as follows:

The step-by-step procedure of the dynamic active sensing server load managing (DASLM) algorithm to obtain the above-mentioned goals is as follows, with a flow chart representation of each step:

Step 1

After establishing the SDN environment, the first step in the algorithm determines which controller is required depending on the selected network topology. In this research article, the single POX controller with the DASLM algorithm manages the load on user-defined network topology. However, using a single, centralized mode-based SDN controller on large data center networks (DCNs) comparing several hundred thousand components or network types mentioned above will lead to more significant latency delays. Eventually, it could result in network failure due to data traffic overloading and bottlenecks [81] issues. The quality parameters in larger DCNs controlled by a single SDN controller will decrease significantly [82]. An experimental study is presented in a research article [83] in which significant DCN data traffic is controlled by a single NOX controller with low-quality parameters (i.e., latency of 10 ms). To combat the higher latency issue in larger SDN-based DCNs, the SDN controller should be used in either a logically centralized or logically distributed arrangement [84,85]. However, no universal technique or method provides the exact formulation to define when the Software-Defined Networking (SDN) is considered “large.” The following fundamental factors can be helpful to explain if the selected SDN-based network is small or large:

(1) Network devices (routers, switches, etc.).
(2) Network infrastructure.
(3) QoS results extraction.
(4) When the controller of an SDN-based network provides greater latency delays in HTTP request handling and indicates that the controller is not performing load management tasks properly. Example of a large SDN-based network:
  (a) A network has a hundred thousand network devices (routers, switches, servers, etc.), a large number of concurrent HTTP requests generating end users, and multiple data centers.
  (b) A network has hundreds of thousands of virtual machines, and their communication is managed through SDN-based applications.
  (c) An SDN network provides services to many end-users covering a sizeable geographical area.

**Figure 5:**
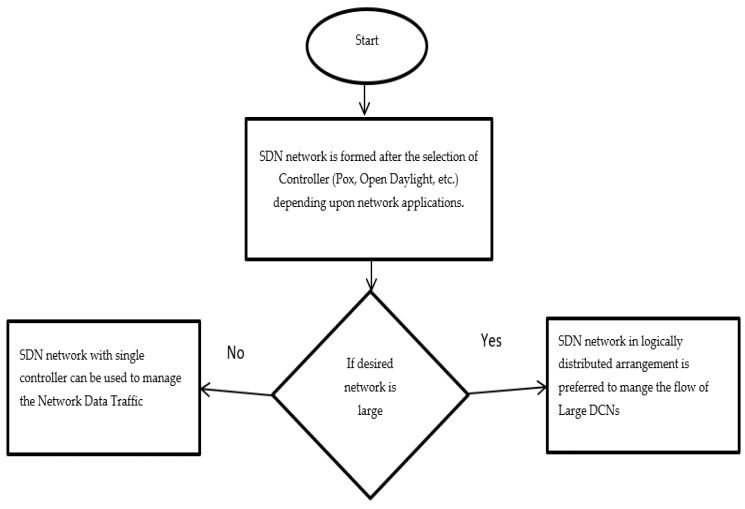
Flow diagram of step #1 in the DASLM algorithm.

To overcome this problem illustrated in research articles [81,82,83], this research article proposes that the large SDN network be divided into the smaller SDN networks, with the controller in a logically distributed controlled environment (to overcome the compatibility issue). Each controller is responsible for their (subdivided) SDN network, and information regarding the tele-traffic details is shared through a communication link (wired or wireless). An explanation of step #1 in the DASLM algorithm is shown in Figure 5.

Step 2

The primary purpose of the proposed technique is to prevent HTTP servers from overloading. After forming the SDN network, the next step in the algorithm is to calculate the number of HTTP requests on each server. Equations (1) and (2) are formulated in the DASLM algorithm to determine each server’s HTTP requests/second (RPS).

Number of requests = Jstep × NPJ × Nuser/sec(1)

(2) $$\mathrm{HTTP}\;\mathrm{requests}/\mathrm{second}\;(\mathrm{RPS})=\frac{Number\;of\;requests}{Total\;duration\;of\;time}$$

RPS represents HTTP requests per second, J_step_ represents the number of steps in the journey, N_PJ_ represents the number of HTTP requests per journey, and N_users/sec_ represents the number of users per second. A journey is defined as an action performed by the user to originate an HTTP request to obtain the required information from a requested server. Sometimes, a simple HTTP request journey involves different steps corresponding to other web pages in addition to the requested web page. During this scenario, additional HTTP requests are generated. Under the influence of the DASLM algorithm, the SDN controller extracts each server’s value of Jstep, NPJ, Nuser/sec, and RPS. Every server has a practical limit for handling HTTP request load; beyond that limit, the server performance decreases and can become unresponsive. In our proposed algorithm (DASLM), we have calculated the HTTP request per second load on each server. To tune the number of HTTP servers (four in our case) to handle the HTTP request load better, we have defined the maximum server load range as (“1000” HTTP requests per second) by using Equation (3).

(3) $$\mathrm{Server}\;\mathrm{load}\;\mathrm{range}=\frac{total\;number\;of\;HTTP\;requests\;generated}{time\;in\;seconds}$$

The HTTP traffic in an SDN-based network is calculated using the Wireshark tool in the Mininet. In our simulation model, “15,000” HTTP requests were generated during 15 s. Using Equation (3), we have set the server load range as (“1000” HTTP requests per second). Beyond this defined range, the server is considered loaded, and the HTTP request flow is assigned to the next available server in the network for load balancing. We have defined this server load range (“1000” HTTP requests per second) as the server load threshold (S_LT_) value. If the number of HTTP requests on the particular server has reached this limit (“1000” HTTP requests per second), then that server is considered loaded. It is removed from the available pool of servers in the SDN network. No new HTTP request is assigned to that server until the RPS value decreases below the defined (S_LT_) value.

No fixed theoretical limit applies universally to all SDN-based networks; one has to conduct load testing to find the most suitable server load range for efficient server load balancing in SDN-based networks. However, we have conducted load testing and monitored server performance (regarding network QoS parameters) to find the best server load theoretical value (“1000” HTTP requests per second in our case).

QoS parameter testing:

The “15,000” HTTP requests were generated from the randomly available twenty-three hosts and forwarded to the POX controller, whose task is to perform the HTTP request load balancing among four servers under the influence of the proposed algorithm (DASLM). As explained earlier, the maximum server load range defined as the server load threshold (S_LT_) value is compared to the RPS value. In this case, to check the authenticity of the S_LT_ value, the network testing is performed in four parts. We have set the server load range to the value of (a) “2000” HTTP request per second, (b) “3000” HTTP requests per second, (c) “3750” HTTP requests per second, calculated from Equation (4), and (d) “1000” HTTP requests per second, calculated from Equation (3).

(4) $$\mathrm{Server}\;\mathrm{load}\;\mathrm{range}=\frac{total\;number\;of\;HTTP\;requests\;generated\;}{total\;of\;HTTP\;server\;avaiable\;in\;the\;network}$$

**Figure 6:**
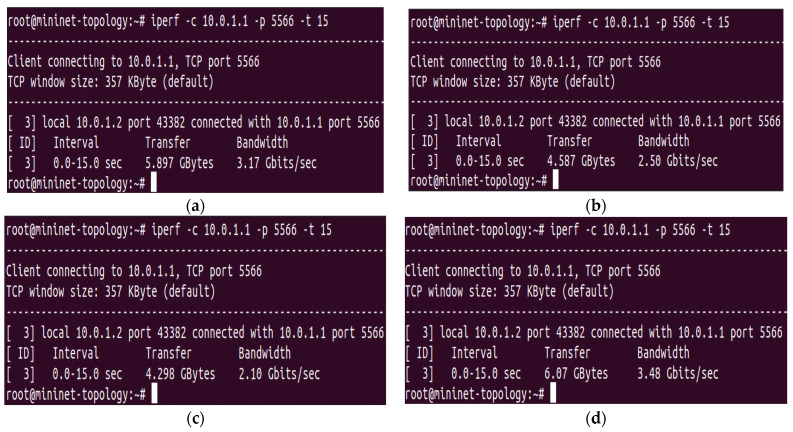
(**a**–**d**) QoS parameters of the interface between the controller and virtual for four load tests. (**a**) QoS parameters of the interface between the controller and virtual terminal using “2000” HTTP per second. (**b**) QoS parameters of the interface between the controller and virtual terminal using “3000” HTTP per second. (**c**) QoS parameters of the interface between the controller and virtual terminal using “3750” HTTP per second. (**d**) QoS parameters of the interface between the controller and virtual terminal using “1000” HTTP per second.

Using the I-Perf utility, the network performance in QoS parameters is measured in all four cases for 15 s. The statistical data (QoS parameters) of all the above four cases are shown in Figure 6.

With reference to Figure 6, the QoS parameters are greater when the maximum server load value is selected using Equation (3). That is why the server load value defined as the S_LT_ value is chosen as “1000” HTTP requests per second. The QoS parameters (in terms of the transfer rate, throughput, and maximum available bandwidth) are summarized in Table 2. B_am_ represents the maximum available bandwidth, T_f_ is the transfer rate, and T_h_ is the throughput.

Conclusion (from four test cases):

Regarding QoS parameters obtained in Table 2, the proposed algorithm’s (DASLM) performance was better at defining the reference HTTP server load (S_LT_) value (“1000” HTTP requests per second) and comparing it with the measured RPS value. If the number of HTTP requests on the particular server reaches this limit (“1000” HTTP requests per second), then that server is considered loaded. It is removed from the available pool of servers in the SDN network, and no new HTTP request is assigned to that server until the RPS value decreases below the defined (S_LT_) value. Further load balancing is performed as mentioned in step 3 of the proposed (DASLM) algorithm.

Step 3

Suppose the load traffic on a certain server exceeds the defined range of the S_LT_ value (as explained in step 2), in that case, the HTTP request is shifted to other servers for equal load sharing depending upon the fulfillment of the following sequential conditions:

IF (condition ==true)

{

The new HTTP request flow is assigned to the server with a smaller RPS load value than the list of available servers in the network.

ELSE

The new HTTP request flow is assigned to the server with less than 60 HTTP requests in the queue (considered a reference value in our case) in addition to the HTTP requests being processed by that server.

ELSE

The new HTTP request flow is assigned to the server with a quicker response time than all other available servers in the network. This task is accomplished by sending an ARP packet to the servers. The server responds to the controller with less latency, and the new HTTP request flow is forwarded to that server.

}

**Figure 7:**
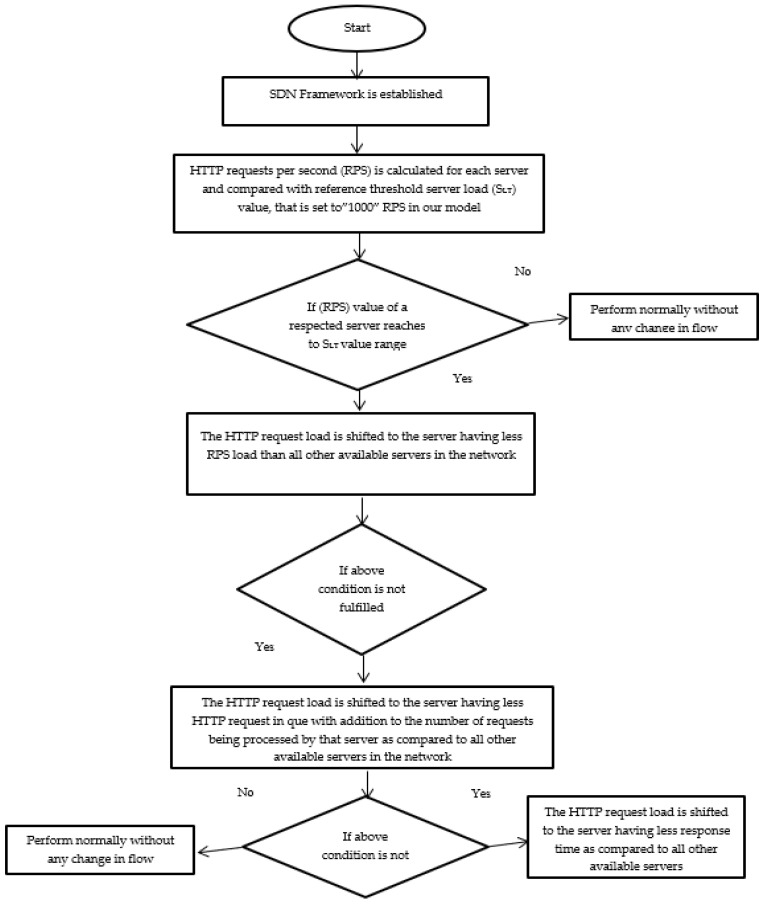
Flow diagram of steps 2 and 3 in the DASLM algorithm.

An explanation of step #2 and step #3 in the DASLM algorithm is shown in Figure 7.

### 3.2. Procedural Steps

The simulation in this manuscript is performed in two portions: (1) without implementing the dynamic active sensing server load management (DASLM) algorithm on the SDN controller and (2) implementing the dynamic active sensing server load management (DASLM) algorithm on the SDN controller.

**Figure 8:**
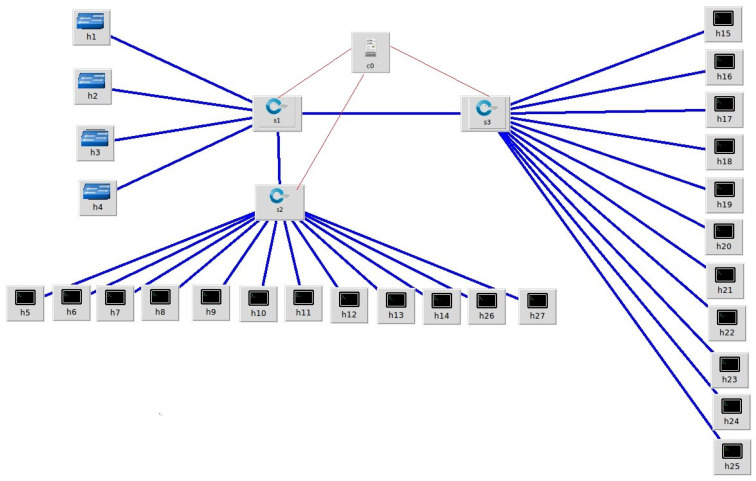
User-defined network topology on Mininet.

Portion 1:

(a) SDN controller (POX) is first switched (up) to the running condition.
(b) The network topology (shown in Figure 8) is drawn on the Mininet graphical interface or can be established by writing a command in the command line interface of Mininet.
(c) Server load (in terms of HTTP requests) is calculated, and based on these calculations, the graph of the QoS parameters is obtained using the I-Perf and Gnu-plot utility.

Portion 2:

(a) The controller (POX) is switched to active mode by running the DASLM algorithm (with details as mentioned in Section 3.1).
(b) The network topology (shown in Figure 8) is drawn on the Mininet graphical interface or can be established by writing a command in the command line interface of Mininet.
(c) Server load (in terms of HTTP requests) is calculated, and based on these calculations, the graph of the QoS parameters is obtained using the I-Perf and Gnu-plot utility.
(d) The comparison is drawn between the QoS parameters results obtained in both portions (1 and 2). However, the QoS parameter results in portion 2 will be far superior to those obtained in portion 1 (the QoS result details are explained in Section 4).

### 3.3. SDN-Based Environment (Lab Setup)

For SDN-based environment creation, we require the following:

(a) Two cores i-7 (HP 15 Dw4029NE, Core i7, 12th Generation, 256 GB SSD, 1 TB HDD, 2 GB NVIDIA MX550 DOS), ten generations with 32 GB RAM each.
(b) With three VMs (virtual machines) on each PC, one is used to run an SDN controller, one for the Mininet topology, and the other for the network performance graph.
(c) Mininet is required to simulate the network along the I-Perf and J-Perf (required for QOS parameter measurement).
(d) P-J-T graph and Gnu-plot utility convert text files in the simulated graph for QOS parameter analysis.
(e) Wireshark tool (for network graphs).
(f) POX Controller (scripted in Python—version: 3.11.4).

Table 3 represents the network parameters to be used in the simulation of a user-defined network in a Mininet environment.

## 4. Simulation Results and Discussion

The network selected for the simulation consisted of one POX controller with twenty-seven hosts and three switches. We converted the first four hosts (h_1_, h_2_, h_3_, and h_4_) to HTTP servers (s_1_, s_2_, s_3_, and s_4_) using the Python command. The network topology is illustrated in Figure 8.

We performed the simulation in two portions: (1) without implementing the dynamic active sensing server load management (DASLM) algorithm on the SDN controller and (2) implementing the dynamic active sensing server load management (DASLM) algorithm on the SDN controller with two cases, (A) normal flow and (B) loaded scenario. The results of the QoS parameters are obtained using the I-Perf utility. In the first portion of the simulation, in which no load management algorithm was loaded on the controller, the remaining twenty-three hosts were randomly used to generate 150 HTTP requests in normal flow, and 15,000 HTTP requests in the loaded scenario case, and they all were sent to four servers (s_1_, s_2_, s_3_, and s_4_). In the second portion, the proposed algorithm technique (DASLM) was implemented on the POX controller using twenty-three hosts randomly; 150 HTTP requests in normal flow and 15,000 HTTP requests in the loaded scenario were generated and sent to the controller, which performs the load management task under the directions of the proposed technique.

### 4.1. (CaseI: Finding QoS Parameters of User-Defined Network Topology without the DASLM Algorithm)

#### 4.1.1. Normal Flow

**Figure 9:**
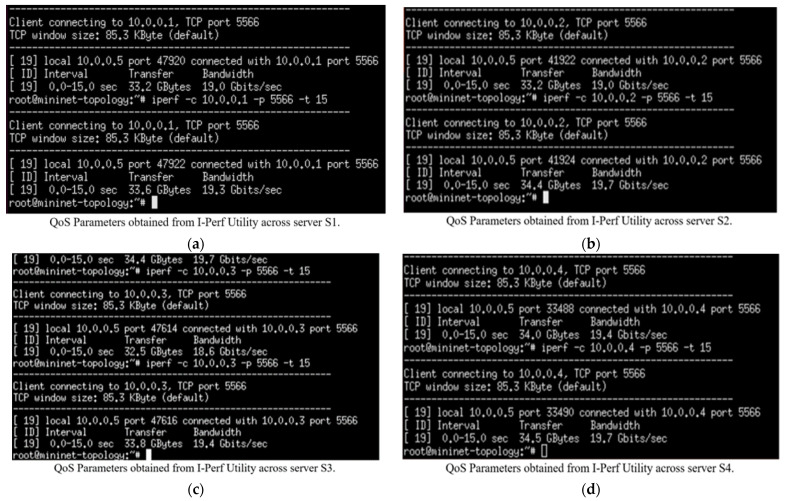
(**a**–**d**) QoS parameters of HTTP servers (s_1_, s_2_, s_3_, and s_4_) under normal flow without the DASLM algorithm through the I-Perf utility.

The maximum available bandwidth, throughput, and transfer rate were calculated for normal flow with 150 HTTP requests on each server. These calculations were performed using the I-Perf utility. The default IP addresses for servers (s_1_, s_2_, s_3_, and s_4_) are (10.0.0.1), (10.0.0.2), (10.0.0.3), and (10.0.0.4), respectively. The statistical data (QoS parameters) fetched from these HTTP servers linked by the I-Perf utility for 15 s are shown in Figure 9.

For a better understanding of the statistical data (in terms of transfer rate, throughput, and maximum available bandwidth) fetched across the four server links shown in Figure 9, they are summarized in Table 4, where B_am_ represents the maximum available bandwidth, T_h_ represents the throughput, and T_f_ represents the transfer rate.

**Figure 10:**
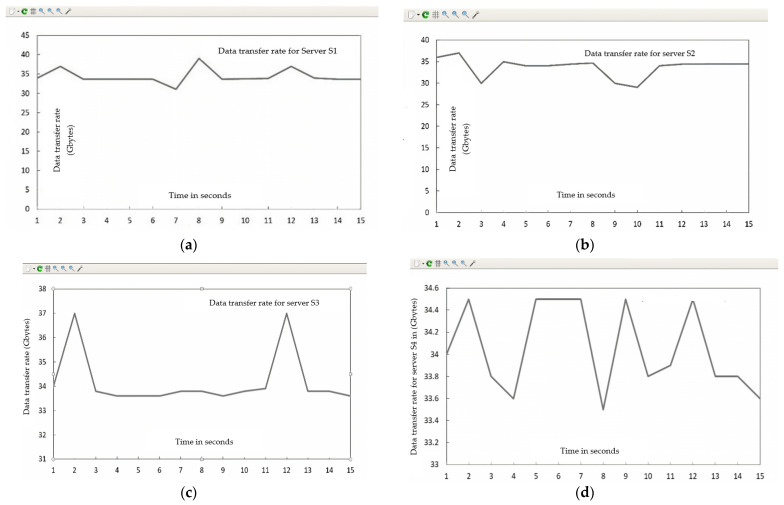
The QoS parameter (transfer rate = T_f_) of HTTP servers under normal flow without implementation of DASLM algorithm. (**a**) (Transfer rate = T_f_) of HTTP server (s_1_). (**b**) (Transfer rate = T_f_) of HTTP server (s_2_). (**c**) (Transfer rate = T_f_) of HTTP server (s_3_). (**d**) (Transfer rate = T_f_) of HTTP server (s_4_).

The Gnu-plot utility represents the maximum available bandwidth and transfer rate across the four HTTP servers in the line graphs. Figure 10 shows the QoS parameters (TCP data transfer rate T_f_) across the servers (s_1_, s_2_, s_3_, and s_4_).

**Figure 11:**
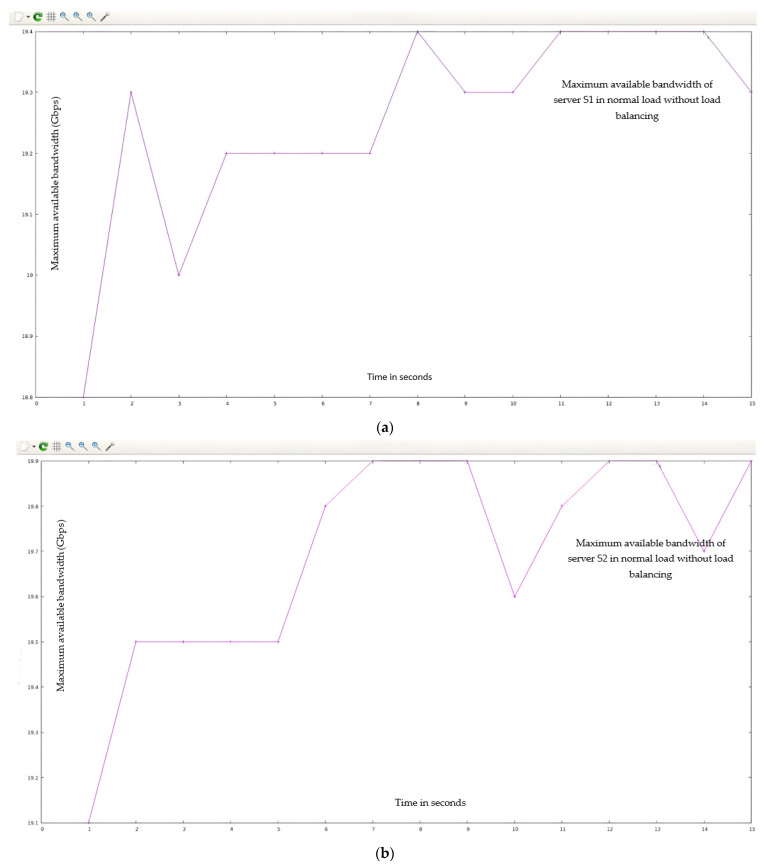

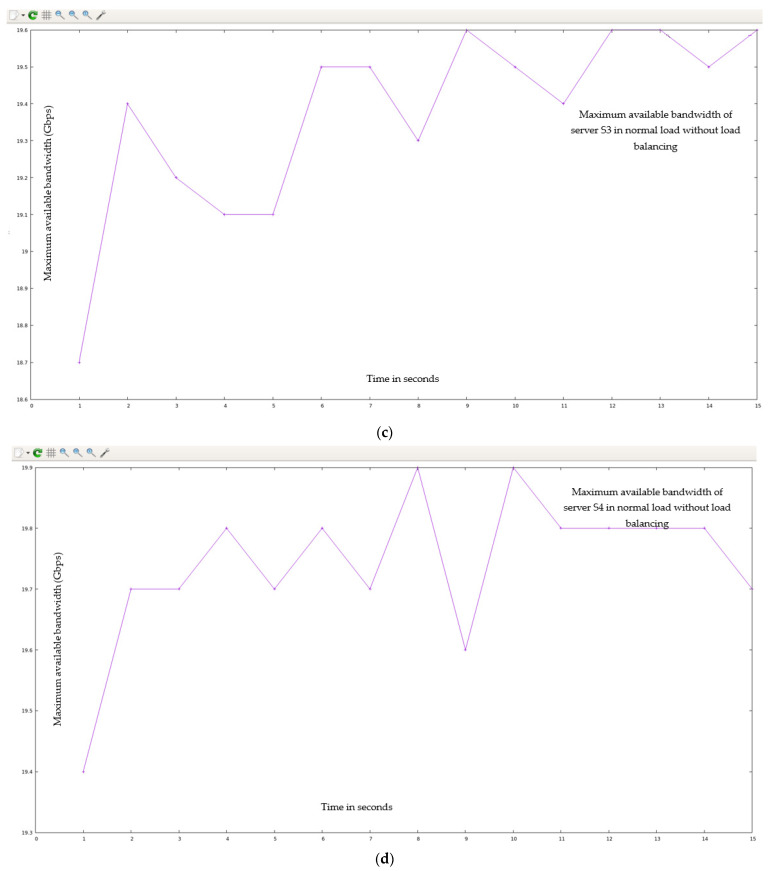
(**a**) QoS parameters (max available bandwidth) of HTTP server (s_1_) under normal flow without implementation of DASLM algorithm. (**b**) QoS parameters (max available bandwidth) of HTTP server (s_2_) under normal flow without implementation of DASLM algorithm. (**c**) QoS parameters (max available bandwidth) of HTTP server (s_3_) under normal flow without implementation of DASLM algorithm. (**d**) QoS parameters (max available bandwidth) of HTTP Server (s_4_) under normal flow without implementation of DASLM algorithm.

Figure 11 shows the QoS parameters (maximum available bandwidth) across the servers (s_1_, s_2_, s_3_, and s_4_).

Summarizing the simulation results obtained from Case I (normal flow):

The maximum available bandwidth, throughput, and transfer rate were calculated for normal flow with 150 HTTP requests on each server. These calculations were performed by fetching data across four server (s_1_, s_2_, s_3_, and s_4_) links for 15 s using the I-Perf utility and Gnu-plot. Referring to Figure 9 and Figure 11, the Gnu-plot displays the maximum available bandwidth of the four servers in the form of a line graph. The maximum available bandwidths of servers s_1_, s_2_, s_3_, and s_4_ with 150 HTTP requests are 19.3 Gbps, 19.7 Gbps, 19.4 Gbps, and 19.7 Gbps, respectively. Referring to Figure 9 and Figure 10, the average transfer rate across the link of servers s_1_, s_2_, s_3_, and s_4_ with 150 HTTP requests are 33.6 Gbytes, 34.4 Gbytes, 33.8 Gbytes, and 34.5 Gbytes, respectively. Equation (5) is used to determine the throughput across the four server links. The throughput of servers s_1_, s_2_, s_3_, and s_4_ with 150 HTTP requests are 17.92 Gbps, 18.34 Gbps, 18.0266 Gbps, and 18.4 Gbps, respectively.

(5) $$Throughput\;\left(Gbps\right)=\frac{Data\;Transfer\;Rate\;in\;Gbytes}{Time\;in\;sec}$$

The I-Perf utility makes one host a client and the other a server to which the HTTP request is forwarded. The I-Perf utility represents the statistical value of the network traffic at the server–client interface each time. The values that I-Perf represents are variable results (data) owing to the ratio of change in the network traffic across the server–client interface. In our case, as shown in Figure 10 and Figure 11, a total of 150 HTTP requests were generated and forwarded to each server (s_1_, s_2_, s_3_, and s_4_) from the randomly available host in a total simulation time of 15 s. Therefore, the data represented by I-Perf in the real-time instance are variable because it takes 15 s for 150 HTTP requests to reach the servers. Hence, the data traffic ratio is different every instant (15 s), so the network traffic constantly changes.

#### 4.1.2. Loaded Scenario

In this experiment, “15,000” HTTP requests were generated by randomly available twenty-three hosts (shown in Figure 8) and only directed to a specific server, s_2_; no requests were generated for the other servers. In this case, the maximum available bandwidth, throughput, and transfer rate are calculated under loaded conditions for server s_2_ (only). These calculations were performed using the I-Perf utility for 15 s. The QoS parameters (transfer rate, throughput, and maximum available bandwidth) fetched across the HTTP server s_2_ under loaded conditions are summarized in Table 5, where B_am_ represents the maximum available bandwidth, T_h_ is the throughput, T_avr_ represents the time of arrival, L represents the latency, %T_f_ represents the percentage drop-in transfer rate compared to normal flow, %L represents the percentage increase in server load compared to normal flow, and T_f_ represents the transfer rate.

**Figure 12:**
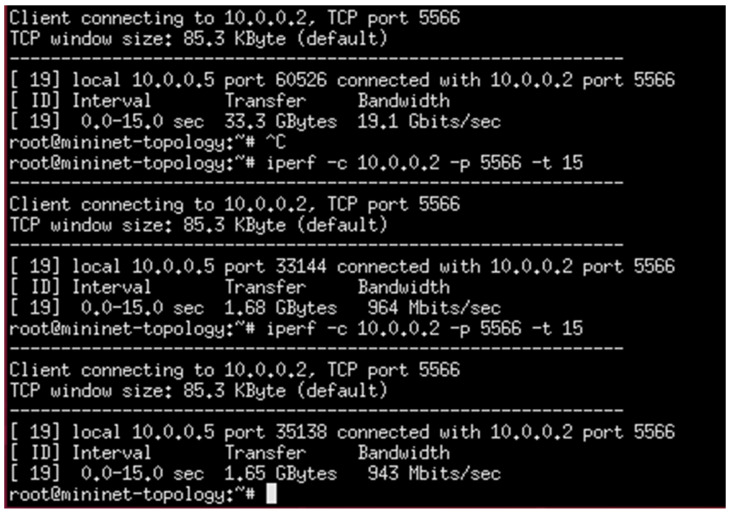
QoS parameters of HTTP server (s2) under a loaded scenario without DASLM through I-Perf utility.

The statistical data (QoS parameters) fetched from these HTTP servers linked by the I-Perf utility for 15 s are shown in Figure 12.

As is evident from the data in Table 5, when all HTTP requests are sent to a specific server without an efficient load-balancing mechanism, the QoS parameters decrease, which results in the degradation of the network efficiency. The %L and %T_f_ explain that when all requests are directed to the specific server, the available bandwidth decreases (19.7 Gbps to 943 Mbps) owing to extra load (with only 4.78% available bandwidth and a load of approximately 95%) on the targeted server which is not shared by the other servers in the network. This causes the targeted server to have a bottleneck condition, and the transfer rate decreases drastically to 1.65 Gbytes, with a decrease in transfer rate of 95.43% compared to normal flow. Implementing the proposed DASLM algorithm based on the aforementioned parameters can achieve high performance. The results are represented in Case II. Figure 12 shows the QoS parameters (maximum available bandwidth and transfer rate) across the targeted server s_2_. The B_am_ and T_f_ values obtained during the normal flow are considered references and compared to the loaded scenario, which is why the %L and %T_f_ values in the first row of Table 5 are marked (X). Equations (6) and (7) were used to find %L (percentage increase in server load compared to normal flow) and %T_f_ (percentage drop-in transfer rate compared to normal flow).

(6) $$\%L=100-\left(\frac{100\times (avaible\;bandwidth\;under\;loaded\;case)}{avaible\;bandwidth\;under\;normal\;flow\;}\right)$$

(7) $$\%T_{f}=100-\left(\frac{100\times \left(transfer\;rate\;under\;loaded\;case\right)}{transfer\;rate\;under\;normal\;flow}\right)$$

**Figure 13:**
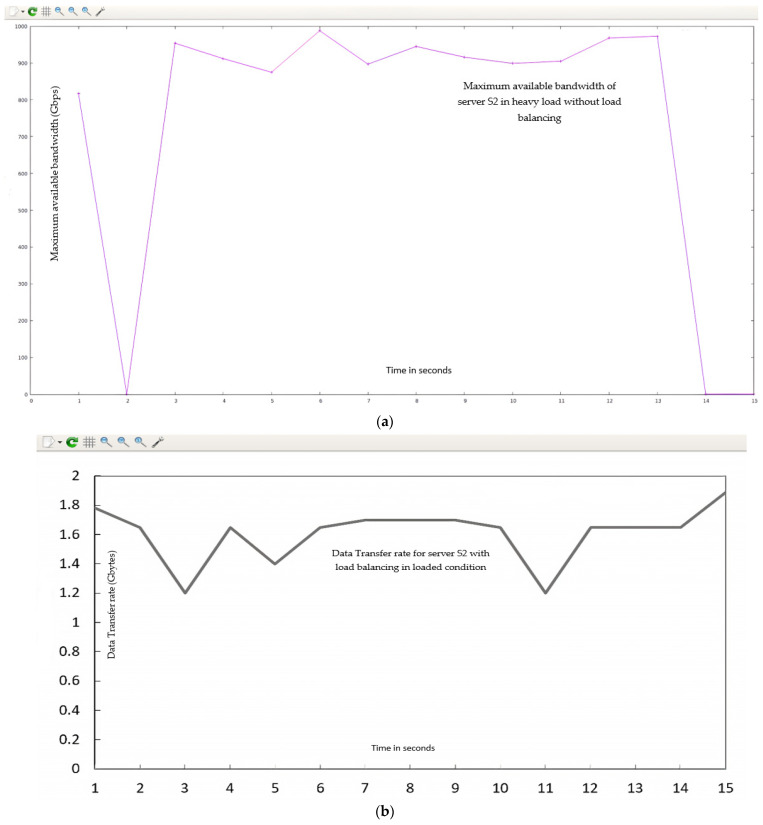
(**a**) QoS parameters (maximum available bandwidth) of targeted server s_2_ under a loaded scenario without DASLM. (**b**) QoS parameter (transfer rate T_f_) of HTTP server (s_2_) under a loaded scenario without implementation of DASLM.

Summarizing simulation results obtained from Case I (loaded flow):

The maximum available bandwidth, throughput, and transfer rate were calculated for a loaded flow, with all “15,000” HTTP requests directed only to the targeted server (s_2_) from randomly available hosts (as shown in Figure 8). These calculations were performed by fetching the data across the server (s_2_) link for 15 s using the I-Perf utility and Gnu-plot. Referring to Figure 12 and Figure 13, the Gnu-plot displays the maximum available bandwidth and transfer rate across the server (s_2_) in a line graph. The maximum available bandwidth of the server (s_2_) with “15,000” HTTP requests is decreased from 19.7 Gbps to 943 Mbps. The transfer rate across the link of server S_2_ is reduced from 34.4 Gbytes to 1.65 Gbytes. Equation (5) was used to find the throughput across the s_2_ links. The throughput of the server (s_2_) is also decreased from 18.34 Gbps to 0.88 Gbps. As no load-balancing algorithm technique is applied to the controller of the SDN, the server load on s_2_ is increased up to 95% (calculated from Equation (6)). The drop-in transfer rate of the server (s_2_) is about 95.43% (calculated from Equation (7)). The ping command was used to find processing delays and latency. The arrival times of 156 packets were calculated. The processing delay is 974.19 ms in the loaded scenario. The latency increases to 12 ms, as mentioned in Table 5.

### 4.2. Case II: Finding QoS Parameters of User-Defined Network Topology with the Implementation of the DASLM Algorithm on an SDN Controller

This portion was divided into two parts to better interpret the results: (1) normal flow and (2) loaded scenario. When the DASLM algorithm script is loaded onto the SDN controller, it acts as a loadmaster and distributes the HTTP request from the host to a pool of available network servers. There is a slight difference in case II compared to the previously discussed case I. Here, HTTP requests are directed to the SDN controller, which performs a load-managing task under the instruction of the DASLM.

#### 4.2.1. Normal Flow

In the case of a normal flow, “150” HTTP requests are directed to the controller, which, under DASLM, fulfills the user HTTP requests. The maximum available bandwidth, throughput, and transfer rate were calculated using the I-Perf utility for 15 s. Here, virtual traffic is generated from one virtual machine, and the controller is designed on the other. As per previous practice, “150” HTTP requests were sent from the virtual machine to the controller loaded with the DASLM algorithm script. The controller IP address is 10.0.1.1, whereas the virtual machine on which the virtual traffic is generated is 10.0.1.2. For a better understanding of the data (in terms of transfer rate, throughput, and maximum available bandwidth) fetched across the link connecting the controller under DASLM and the virtual machine, where virtual traffic is generated as HTTP requests, they are summarized in Table 6, where B_am_ represents the maximum available bandwidth, T_h_ represents the throughput, and T_f_ represents the transfer rate.

**Figure 14:**
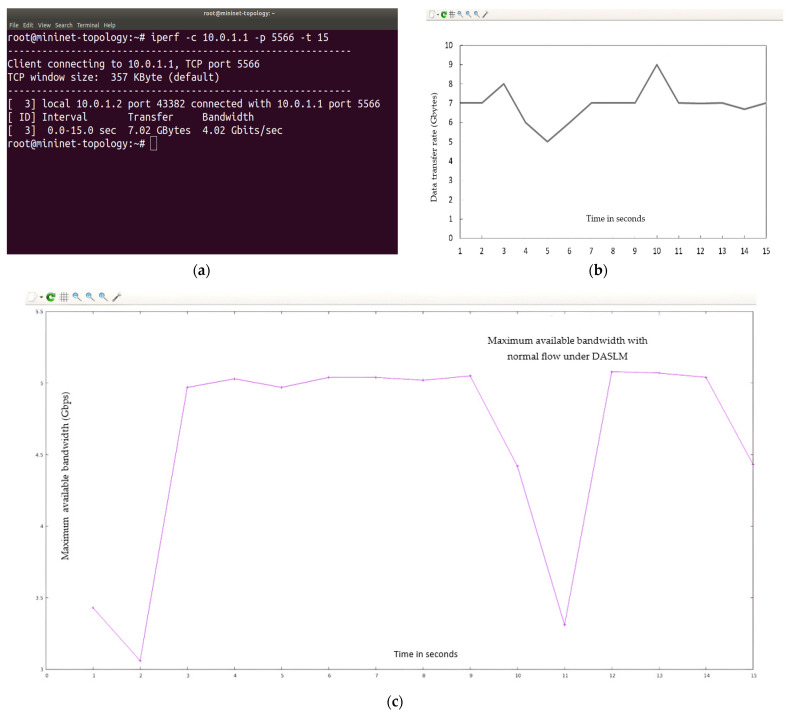
(**a**–**c**) show the QoS parameters under normal flow with the implementation of the DASLM algorithm. (**a**) represents statistical information about QoS parameters on the Mininet terminal. (**b**) represents the transfer rate (T_f_). (**c**) represents the maximum available bandwidth.

The Gnu-plot represents the maximum available bandwidth and transfer rate in the line graphs. Figure 14 illustrates the QoS parameters (data transfer rate and maximum available bandwidth).

Summarizing simulation results obtained from Case II (normal flow):

In the case of a normal flow, 150 HTTP requests are directed to the controller, which, under DASLM, fulfills the user HTTP requests. The maximum available bandwidth and transfer rate calculations were performed using the I-Perf utility for 15 s. Referring to Figure 14, the Gnu-plot displays the maximum available bandwidth and transfer rate across the link between the controller and virtual machine in a line graph. The maximum available bandwidth is 4.02 Gbps. The transfer rate is 7.02 Gbytes. Equation (5) was used to find the throughput across the link between the DASLM-based controller and the virtual machine. The throughput is 3.744 Gbps.

#### 4.2.2. Loaded Scenario

**Figure 15:**
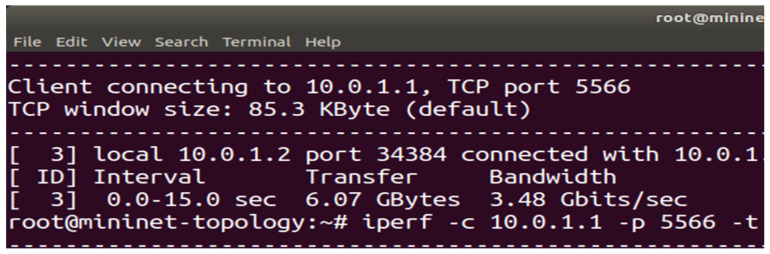
QoS parameters of the interface between the controller and virtual machine under DASLM with the loaded scenario.

In this case, “15,000” HTTP requests are generated on the interface between the controller and the virtual machine. The maximum available bandwidth, throughput, and transfer rate were calculated under loaded conditions. These calculations were performed using the I-Perf utility for 15 s. Summary of statistical data obtain from Iperf utility is shown in Figure 15.

The QoS parameters (in terms of the transfer rate, throughput, and maximum available bandwidth) under loaded conditions are summarized in Table 7, where B_am_ represents the maximum available bandwidth, T_h_ is the throughput, T_avr_ is the time of arrival, L is the latency, %T_f_ represents the percentage drop-in transfer rate as compared to normal flow, %L represents the percentage increase in server load compared to normal flow, and T_f_ represents the transfer rate. Equations (8) and (9) are used to find the maximum available bandwidth percentage (%B_max_) and achievable transfer percentage (%T_af_), respectively.

(8) $$\%B_{max}=\left(\frac{100*(avaible\;bandwidth\;under\;loaded\;case)}{avaible\;bandwidth\;under\;normal\;flow\;}\right)$$

(9) $$\%T_{af}=\left(\frac{100*(transfer\;rate\;under\;loaded\;case)}{transfer\;rate\;under\;normal\;flow}\right)$$

As is evident from the data in Table 7, by implementing the proposed DASLM algorithm based on the parameters mentioned above, very high-performance QoS parameters are achieved when all HTTP requests (“15,000” in our case) are directed to the controller under DASLM, the available bandwidth increases from 943 Mbps to 3.48 Gbps, along with an increase in the transfer rate from 1.65 Gbytes to 6.07 Gbytes. %T_f_ represents the percentage drop-in transfer rate compared with the normal flow, and % L represents the percentage increase in server load. The factors %L and %T_f_ are also reduced from 95% to 13.43% and 95.43% to 13.53%, respectively, compared to case I without DASLM implementation. The latency and packet arrival time are also reduced, resulting in a decreased processing delay. With the implementation of the DASLM algorithm, efficiency in terms of maximum available bandwidth is increased from 4.786% (in case I) to 86.57%. The transfer efficiency with throughput also rose from 4.65% to 86.47%.

**Figure 16:**
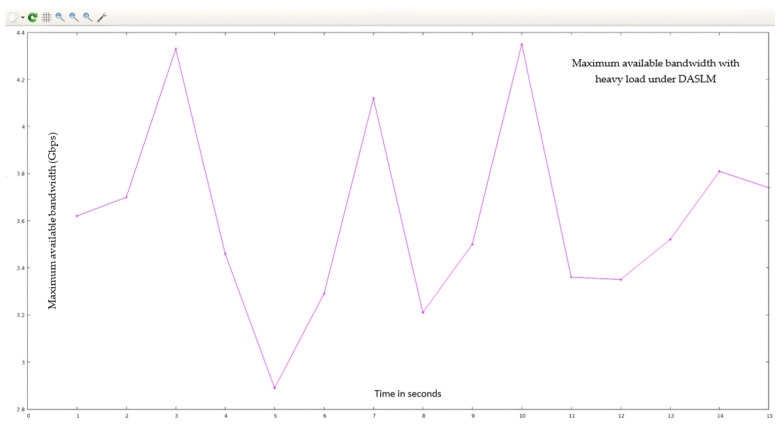
Represents the QoS parameter (maximum available bandwidth) with the loaded scenario with DASLM.

**Figure 17:**
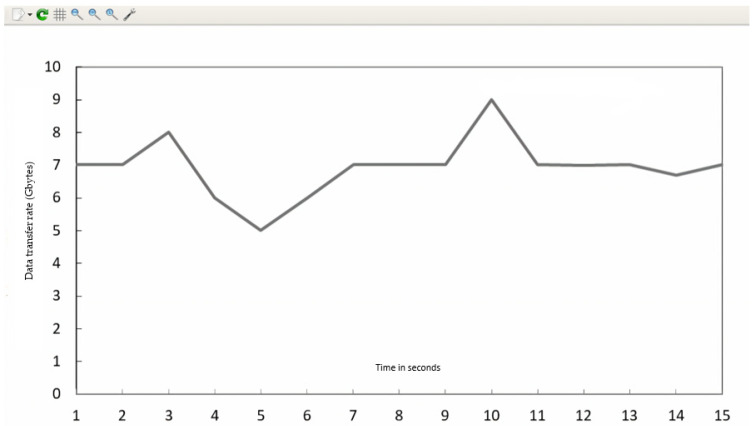
Represents the QoS parameter (transfer rate) with the loaded scenario with DASLM implementation.

The B_am_ and T_f_ obtained during the normal flow are considered references, and their values were compared to the loaded scenario, which is why %L and %T_f_ values in the first row of Table 7 are marked (X). The Gnu-plot utility determines the maximum available bandwidth, throughput, and transfer rate graphs. Figure 16 represents the QoS parameter (maximum available bandwidth). Figure 17 illustrates the QoS parameter (transfer rate).

Summarizing simulation results obtained from Case II (loaded flow):

In the case of a loaded flow, “15,000” HTTP requests are directed to the controller, which, under DASLM, fulfills the user HTTP requests. The maximum available bandwidth, throughput, and transfer rate calculations were performed using the I-Perf utility for 15 s. Referring to Figure 16 and Figure 17, the Gnu-plot displays the maximum available bandwidth and transfer rate across a link between the controller and the virtual machine in the form of a line graph. The maximum available bandwidth is increased to 3.48 Gbps, compared to the results obtained in Section 4.1.2. The transfer rate is also increased up to the level of 6.07 Gbytes in comparison with the simulation results of Section 4.1.2. Equation (5) was used to find the throughput across the link between the DASLM-based controller and the virtual machine. The throughput is also increased up to the level of 3.23 Gbps. Table 7 shows that with the implementation of the proposed technique, %L (calculated from Equation (6)) has decreased from 95% (without DASLM implementation) to 13.43% (with DASLM implementation), and %T_f_ (calculated from Equation (7)) has also decreased from 95.43% to 13.53%. Ping command was used to determine the latency. The latency decreased from 12 ms (without DASLM implementation) to 0.87 ms (with DASLM implementation).

### 4.3. Comparative Analysis of DASLM with Traditional Server Load-Balancing Methods

To prove the effectiveness of the proposed (DASLM) algorithm, the comparative analysis is conducted with the method discussed in research articles [70,71]. Table 1 explains the fruitful effects of the proposed algorithm (DASLM) compared to the other research methods. The two test cases (Case A and Case B) were conducted to judge the performance of the proposed algorithm. In Case A, the research method of article [70] (using the least server response method) is applied to the SDN controller for server load balancing, and QoS parameters are extracted. In Case B, the research method of article [71] (using the traditional Round-Robin method) is used for the SDN controller for server load balancing, and QoS parameters are extracted.

**Figure 18:**
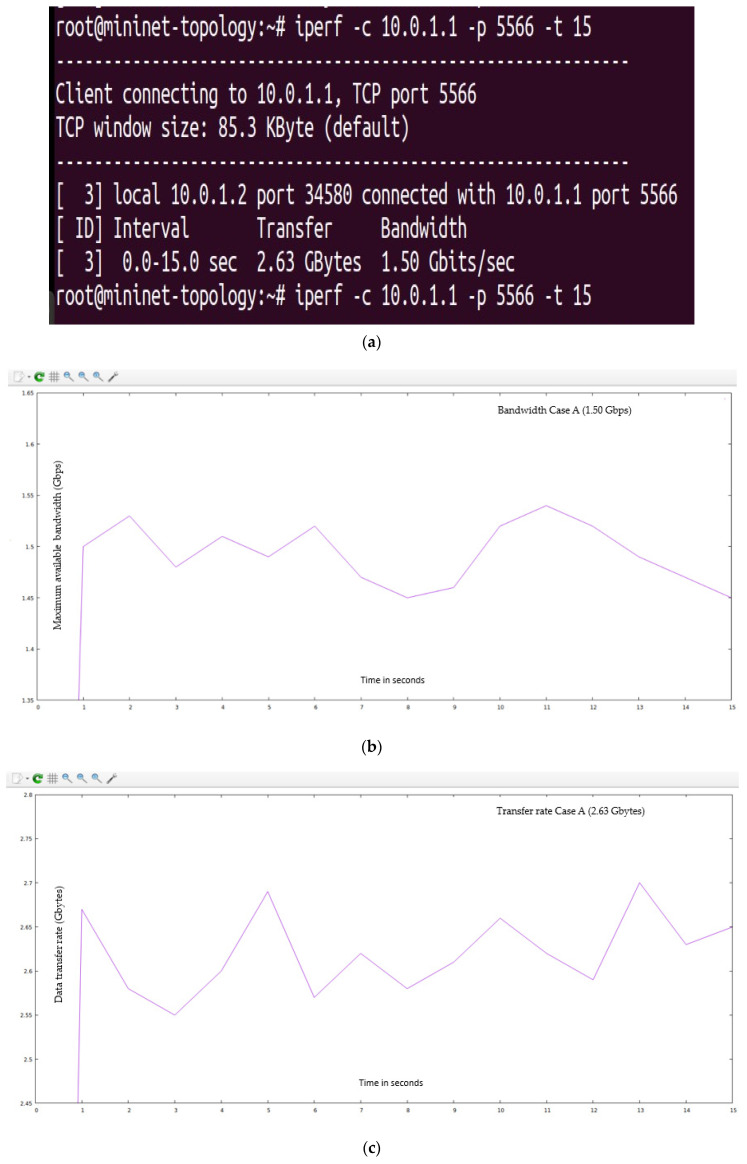
(**a**) Summary of statistical data obtain from Iperf utility for test Case A. (**b**) QoS parameter (maximum available bandwidth) for test Case A (using the least server response method). (**c**) QoS parameter (transfer rate) for test Case A (using the least server response method).

Case A (using the least server response method):

The “15,000” HTTP requests were generated from the randomly available twenty-seven hosts (user-defined network topology is shown in Figure 8) and forwarded to the POX controller. In this case, we have not defined the server load range of 1000 HTTP requests per second but instead measured the response time of HTTP servers in a user-defined network (as shown in Figure 8), as suggested in the research article [70]. This task is accomplished by sending an ARP packet to the servers. The server responds to the controller with less latency, and the new HTTP request flow is forwarded to that server. The maximum available bandwidth and transfer rate was calculated using the I-Perf utility for 15 s. The statistical data (QoS parameters) are shown in Figure 18a. Figure 18b,c represent the bandwidth and transfer rate of the test Case A (with the implementation of the research technique mentioned in the article [70] using the least server response method).

**Figure 19:**
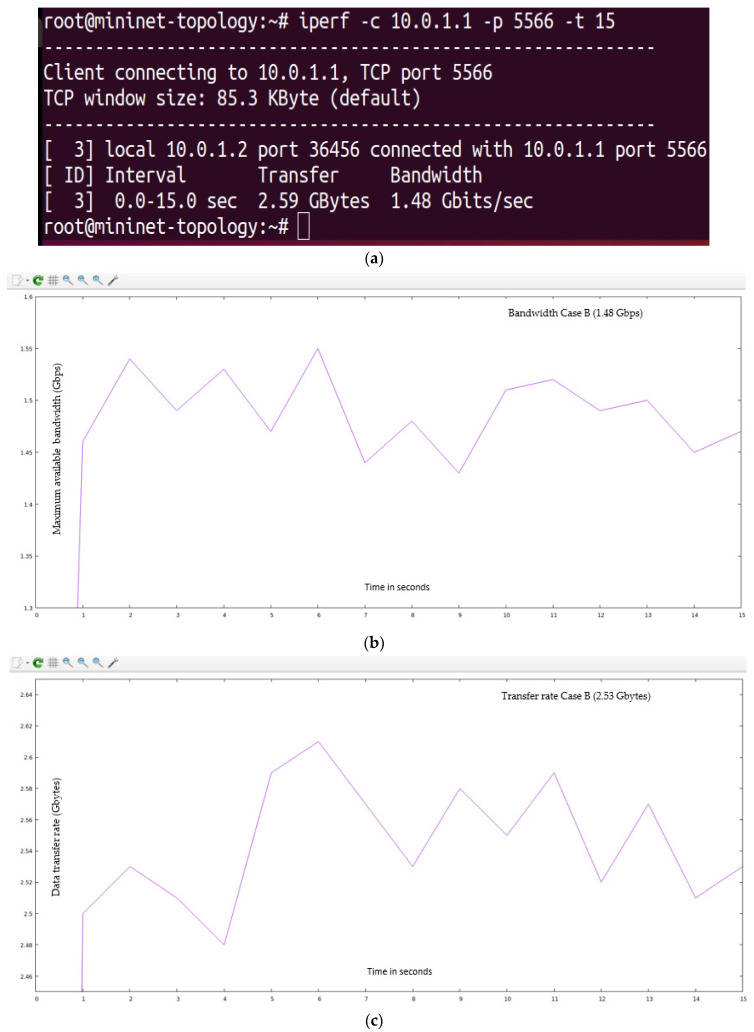
(**a**) Summary of statistical data obtain from Iperf utility for test Case B. (**b**) QoS parameter (maximum available bandwidth) for test Case B (using the traditional Round-Robin method). (**c**) QoS parameter (transfer rate) for test Case B (using the traditional Round-Robin method).

Case B (using the traditional Round-Robin method):

The “15,000” HTTP requests were generated from the randomly available twenty-seven hosts (user-defined network topology is shown in Figure 8) and forwarded to the POX controller. In this case, we have not defined the server load range of 1000 HTTP requests per second but instead applied the traditional Round-Robin approach in a user-defined network (as shown in Figure 8), as suggested in the research article [71]. The I-Perf utility is used to find the B_am_ and T_f_. The statistical data (QoS parameters) are shown in Figure 19a.

Figure 19b,c represent test Case B’s bandwidth and transfer rate (with the implementation of the research article [71] using the traditional Round-Robin method).

A comparison of QoS results obtained from Case A and B with the proposed algorithm (DASLM):

Table 8 summarizes the QoS parameters obtained in Cases A and B with the proposed algorithm (DASLM).

**Figure 20:**
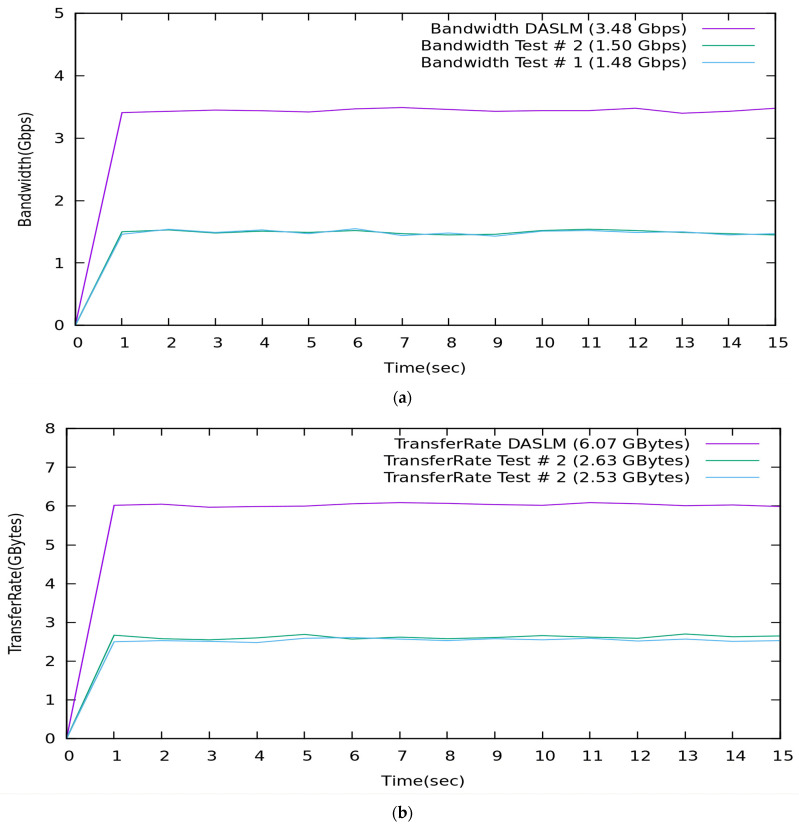
(**a**) Comparison of DASLM with traditional load-balancing method regarding QoS parameter (maximum available bandwidth). (**b**) Comparison of DASLM with traditional load-balancing method regarding QoS parameter (transfer rate).

Figure 20 illustrates the comparative analysis of QoS parameters (maximum available bandwidth and transfer rate).

A Summary of the Comparative Analysis:

With reference to Table 8, the QoS parameters verify that the maximum available bandwidth, throughput, and transfer rate of a user-defined network are improved by implementing the proposed technique in a real-time controlled SDN environment. The performance of the proposed algorithm (DASLM) is far superior to the method mentioned in the research articles [70,71].

## 5. Conclusions

In this study, we achieved the desired results by enhancing the QoS parameters of the HTTP server-based telecom network with the implementation of the proposed server load-balancing technique in a real-time controlled SDN environment, which is the DASLM (dynamic active sensing load managing) algorithm. The simulation in this manuscript was performed in two parts for 15 s: (1) QoS parameter analysis without implementing the DASLM algorithm; (2) QoS parameter analysis with the implementation of the DASLM algorithm. QoS analyses using I-Perf and Gnu-plot utility in the above-mentioned cases were performed based on two scenarios (normal flow with 150 HTTP requests and loaded flow with “15,000” HTTP requests). The QoS parameters in normal flow (with negligible load on the network servers) are considered a reference value to determine the network’s performance in loaded conditions (“15,000” HTTP requests in our simulation model). With the implementation of the proposed technique (DASLM), the QoS parameters (B_am_, T_f_, T_h_, and L) have increased from 943 Mbps to 3.48 Gbps, 1.65 Gbytes to 6.07 Gbytes, 0.88 Gbps to 3.23 Gbps, and 12 ms to 0.87 ms, respectively, under loaded conditions (“15,000” HTTP requests). The maximum available bandwidth percentage (%B_max_) has increased from 4.78% (without DASLM implementation in the loaded scenario) to 86.57% (with DASLM implementation in the loaded scenario). The achievable transfer rate percentage (%T_af_) has also increased from 4.65% (without DASLM implementation in the loaded scenario) to 86.47% (with DASLM implementation in the loaded scenario). These QoS parameters verify that the maximum available bandwidth, throughput, and transfer rate are improved by the implementation of the proposed method (DASLM). For the authenticity of the proposed algorithm, the QoS results obtained from DASLM were compared with the QoS results obtained from the traditional server load-balancing algorithm: (a) server load balancing by calculating the least server response time method and (b) server load balancing by the traditional Round-Robin method; however, the values of the QoS parameters (B_am_, T_f_, T_h_, and L) in the proposed algorithm (DASLM) were far superior to the traditional load-balancing methods and prove the effectiveness of the proposed technique. For future work, the proposed algorithm can be applied to the hierarchical, logically distributed SDN controller environment for server load management, where the whole network is divided into local domains. Each domain will have its controller work under the control parameters of the route and universal controller.

## Figures and Tables

**Table 1:** Comparison between proposed algorithm (DASLM) and traditional load-balancing techniques.

References	Improvement in Network with the Author’s Technique	Limitation	Comparison with the Proposed Algorithm (DASLM)
S. Sathyanarayana et al. [69]	The authors combine the ant colony algorithm with the dynamic flow algorithm. The less-loaded server is found with the dynamic flow algorithm, and the shortest path to the less-loaded server is found using the ant algorithm technique.	In this paper, the latency is reduced. However, combining two algorithms and running them simultaneously extensively uses computer resources, memory, and bandwidth.	The proposed algorithm (DASLM) is a single active sensing dynamic algorithm that balances the load on HTTP servers in the SDN network by calculating their HTTP request load. If the HTTP request load of any server exceeds the range of the server load threshold (S_LT_) value, the load is shifted to the server with less HTTP request load. However, if the above condition is not met, HTTP requests are forwarded to the server with a quicker response time. As a result, the transfer rate, available bandwidth, and throughput are increased, and there are no overhead issues.
H. Zhong et al. [70]	In this research paper, the HTTP request flow is managed based on server response time calculations.	Processing delays are reduced only.	The proposed algorithm (DASLM) not only balances the load on HTTP servers in the SDN network by calculating the response time by sending ARP packets but also selects the optimum server by (a) calculating RPS and comparing the RPS value with the reference threshold S_LT_ value and (b) finding the number of HTTP requests in the queue to be processed by the respective servers of the SDN network.
Hamed et al. [71]	The (HTTP request) load among different servers is balanced using the traditional Round-Robin method.	This method is simple and easy to implement and distributes the HTTP request load among different servers in sequential order. This method has greater limitations in large SDN networks with heavy data flow.	The proposed algorithm (DASLM) is more advanced than the method adopted for load balance [52]. In the proposed method, the optimum server for better managing HTTP request flow is selected based on response time calculation, calculation of RPS and comparison with threshold S_LT_ value, and finding the number of HTTP requests in the queue of respective servers to be processed.
Arahunashi et al. [72]	The (HTTP request) load among different servers is balanced by calculating each server’s maximum available bandwidth.	The throughput and response time calculation is not considered.	The DASLM algorithm performs load balancing among different available servers based on (a) maximum available bandwidth (by calculating the server load), (b)response time, and (c) processing delays.
Kaur et al. [73]	The authors use a direct routing algorithm that directs the server’s response to the host without passing through the load balancer. With this method, the author has claimed a decrease in latency.	The flow control is very much compromised.	In the DASLM algorithm, the RPS value of each server for every flow is calculated, and then, based on comparisons with S_LT_ value, the load is shared among different servers.
Kavana et al. [74]	The authors use a flood light controller, and the link path cost calculation is performed with the shortest path first.	HTTP request load is balanced among different available servers based on link cost optimization and no real-time traffic flow sensing.	DASLM performs real-time HTTP request flow sensing and distributes the HTTP request load among different available servers based on calculations performed for every flow.
Hamed et al. [75]	Comparison of Raspberry-Pi-based network and the network formed on Mininet.	The result claimed by the authors is that the SDN-based network has better performance in server load balancing.	The DASLM is implemented in a Mininet environment with a POX controller.
S. Ejaz et al. [76]	The authors propose using two controllers (master and slave). All copies of files regarding flow management in the network are saved on the master controller so that if the controller fails, other controllers manage the flow.	A logically centralized environment requires tight synchronization.	DASLM proposes the use of a logically distributed environment.
H.Gasmelseed et al. [77]	In this study, the authors propose the use of two controllers. One controller controls the TCP flow, while the other manages the UDP flow and shares files at the end of every flow so that if the controller fails, other controllers manage the flow.	A logically centralized environment requires tight synchronization.	DASLM proposes the use of a logically distributed environment. In this arrangement, every controller manages the flow of their subdivided network.
N.T. Hai et al. [78]	The data traffic is distributed into two categories: (1) critical time traffic and (2) non-critical time traffic, and in the case of congestion, the critical time traffic is given priority.	Minimized data transmission with a greater packet drop ratio.	In the DASLM algorithm, every traffic flow is given equal importance, and real-time HTTP request load calculations manage the flow.
M.L.Chiang et al. [79]	In this research article, the authors use a flood light controller with dynamic load balancing to reach the under-utilized server among different servers available in the network. The HTTP request load is shifted to the server with less RPS (HTTP request per second) load.	No work is conducted regarding response time.	The DASLM algorithm has the advanced feature of computing the server response time and finding the number of HTTP requests in the queue to be processed by the respective server.
H.Zhong et al. [80]	This paper draws a comparison between static and dynamic scheduling algorithms.	The dynamic scheduling algorithm has better flow characteristics.	DASLM is the active sensing load-balancing algorithm that performs real-time calculations to distribute the HTTP request load uniformly among network servers.

**Table 2:** Comparison of QoS parameters in all four tests obtained from I-Perf utility.

Load Testing	Time	B_am_	T_h_	T_f_ (in 15 s)
Test #4 (with 1000 HTTP requests per second)	0–15 s	3.48 Gbps	3.23 Gbps	6.07 Gbytes
Test #3 (with 3750 HTTP requests per second)	0–15 s	2.10 Gbps	2.29 Gbps	4.298 Gbytes
Test #2 (with 3000 HTTP requests per second)	0–15 s	2.50 Gbps	2.44 Gbps	4.587 Gbytes
Test #1 (with 2000 HTTP requests per second)	0–15 s	3.17 Gbps	3.14 Gbps	5.897 Gbytes

**Table 3:** Network parameters to be used in the simulation of a user-defined network in a Mininet environment.

Parameters	Descriptions	Values
T in sec	Total simulation time in sec	0–15
B_am_	Maximum available bandwidth in Gbps	Value to be calculated by I-Perf utility during both cases: (1) normal flow and (2) loaded flow
T_h_	Throughput in Gbps	Value to be calculated by I-Perf utility during both cases: (1) normal flow and (2) loaded flow
T_f_	Transfer rate in G-bytes	Value to be calculated by I-Perf utility during both cases: (1) normal flow and (2) loaded flow
S_LT_ value	Server load threshold value	1000 (RPS) is chosen as the reference value to compute the server load
L	Latency in ms	Value to be calculated by I-Perf utility during both cases: (1) normal flow and (2) loaded flow
RPS	Requests per second	150 RPS during case (1) normal flow and 15,000 during case (2) loaded flow
%T_F_	Percentage decrease in transfer rate	Value to be calculated by I-Perf utility during both cases: (1) normal flow and (2) loaded flow
%L	Percentage increase in server load	Value to be calculated by I-Perf utility during both cases: (1) normal flow and (2) loaded flow
% B_max_	Maximum available bandwidth percentage	Value to be calculated by I-Perf utility during both cases: (1) normal flow and (2) loaded flow
%T_af_	Achievable transfer rate percentage	Value to be calculated by I-Perf utility during both cases: (1) normal flow and (2) loaded flow

**Table 4:** QoS parameters obtained from I-Perf utility (without DASLM).

List of HTTP Servers	Time in s	B_am_	T_h_	T_f_ (in 15 s)
Server#1	0–15 s	19.3 Gbps	17.92 Gbps	33.6 Gbytes
Server#2	0–15 s	19.7 Gbps	18.34 Gbps	34.4 Gbytes
Server#3	0–15 s	19.4 Gbps	18.0266 Gbps	33.8 Gbytes
Server#4	0–15 s	19.7 Gbps	18.4 Gbps	34.5 Gbytes

**Table 5:** Comparison of QoS parameters (with normal and loaded flow) obtained from I-Perf utility (without DASLM).

List of HTTP Servers	Time	B_am_	T_h_	T_f_ (in 15 s)	156 Packets T_avr_ (ms)	L (ms)	%T_f_	%L
S2 (Normal Flow)	0–15 s	19.7 Gbps	18.34 Gbps	34.4 Gbytes	155,790.81	0.299	X	X
S2 (Loaded Scenario)	0–15 s	943 Mbps	0.88 Gbps	1.65 Gbytes	156,765	12	95.43%	95%

**Table 6:** QoS parameters obtained from I-Perf utility with DASLM.

Interface	Time in s	B_am_	T_h_	T_f_ (in 15 s)
The link between the controller and the HTTP request generator virtual machine	0–15 s	4.02 Gbps	3.744 Gbps	7.02 Gbytes

**Table 7:** Comparison of QoS parameters obtained from case I and case II using I-Perf utility.

Interface/Servers	Time	B_am_	T_h_	T_f_ (in 15 s)	Available Bandwidth Percentage	Achievable Transfer Rate (%)	156 Packets T_avr_ (ms)	L (ms)	%T_f_	%L
(Normal Flow) with DASLM	0–15 s	4.02 Gbps	3.744 Gbps	7.02 Gbytes	X	X	790.81	0.2	X	X
(Loaded Scenario) with DASLM	0–15 s	3.48 Gbps	3.23 Gbps	6.07 Gbytes	86.57%	86.47%	865.67	0.87	13.53%	13.43%
(Loaded Scenario) without DASLM	0–15 s	943 Mbps	0.88 Gbps	1.65 Gbytes	4.78%	4.65%	156,765	12	95.43%	95%

**Table 8:** Comparison of QoS obtained from I-Perf utility.

Method Used	Time	B_am_	T_h_	T_f_ (in 15 s)
QoS parameters with DASLM Algorithm.	0–15 s	3.48 Gbps	3.23 Gbps	6.07 Gbytes
Case B	0–15 s	1.48 Gbps	1.38 Gbps	2.59 Gbytes
Case A	0–15 s	1.50 Gbps	1.40 Gbps	2.63 Gbytes

## Data Availability

Data are available from the corresponding author and can be provided upon appropriate request.
